# Challenges for Diagnosis of Malaria and Neglected Tropical Diseases in Elimination Settings

**DOI:** 10.1155/2015/270756

**Published:** 2015-11-05

**Authors:** Stephan Karl, Malcolm K. Jones, Lucía Gutiérrez, Brioni Moore, Eline Kattenberg, Marcus Lacerda

**Affiliations:** ^1^Population Health and Immunity, Walter and Eliza Hall Institute of Medical Research, Parkville, VIC 3052, Australia; ^2^Department of Medical Biology, The University of Melbourne, Melbourne, VIC 3052, Australia; ^3^School of Veterinary Science, The University of Queensland, Brisbane, St. Lucia, QLD 4072, Australia; ^4^Departamento de Biomateriales y Materiales Bioinspirados, Instituto de Ciencia de Materiales de Madrid, 28049 Madrid, Spain; ^5^School of Medicine and Pharmacology, The University of Western Australia, Perth, WA 6009, Australia; ^6^Vector-Borne Infectious Diseases Research Unit, Papua New Guinea Institute of Medical Research, Madang 511, Madang Province, Papua New Guinea; ^7^Fundação de Medicina Tropical Dr. Heitor Vieira Dourado and Instituto de Pesquisa Leônidas e Maria Deane, Fiocruz, 69077-000 Manaus, AM, Brazil

Neglected tropical diseases (NTDs) represent a significant health burden for many countries in the developing world. Currently, there are 17 NTDs prioritized by the World Health Organization (WHO). These NTDs are endemic in 149 countries and affect an estimated 1.4 billion people. NTDs therefore also constitute a very significant burden on the already strained healthcare systems and the economies of many developing countries [1]. To complicate matters, there is not much interest in the development of new diagnostic tools for these diseases. Malaria, for instance, even not formally considered a neglected disease, poses many challenges in terms of the diagnosis of submicroscopic parasitemia [2], which seems to sustain the transmission of the disease in low endemic areas.

Many countries are facing scenarios where transmission of an NTD has been reduced to low levels. In addition, many NTDs cause asymptomatic infections, making the identification of residual transmission foci of these diseases a challenging task. Thus, in these very low transmission settings, with often undetectably low individual pathogen burdens, diagnostic challenges are considerable and the need for developing better techniques and strategies for diagnosis and epidemiological surveillance is strikingly evident.

In this special issue, authors contributed several reviews and original research papers describing current diagnostic challenges for African sleeping sickness, human cystic echinococcosis, dengue and chikungunya fevers, and schistosomiasis.

The development of reliable, highly sensitive, and species specific molecular techniques such as loop-mediated isothermal amplification (LAMP), which are potentially high throughput and more easily adaptable to remote field conditions, may allow for more accurate diagnosis and epidemiological surveillance of a number of NTDs such as* Trypanosoma brucei gambiense* (J. M. Kagira et al.). Particularly noninvasive molecular techniques, for example, approaches using saliva or urine samples, as discussed in several papers in this issue, would constitute significant progress over current techniques relying on microscopy using blood samples or lymph node aspirates (*T. b. gambiense*), which are often not sensitive enough to provide definitive diagnosis. J. Bonnet et al. discuss these issues and review current developments for field-deployable human African trypanosomiasis diagnosis.

The need for the development of reliable serological markers for the detection of infection with* Echinococcus granulosus*, a highly neglected zoonotic tropical helminth infection, is discussed in the contribution of C. Sánchez-Ovejero et al. Reliable serology tools not only could enable better confirmation of ultrasound-based diagnosis of this disease but also could be used in cross-sectional surveys in order to obtain more accurate estimates of the burden of human cystic echinococcosis and other NTDs such as schistosomiasis [2].

The clinical differentiation of dengue and chikungunya virus infections is a particular challenge reviewed in the contribution of S. K. Mardekian and A. L. Roberts. Both viruses result in similar clinical symptoms, especially early in the infection. However outcomes and management strategies for dengue and chikungunya are very different. With the increasing global numbers of dengue and chikungunya infections, and with outbreaks occurring more frequently in southern Europe, northern Australia, and Florida, better understanding of the distinguishing features of these infections is critical [3].

The diagnosis of schistosomiasis is challenging as the number of eggs in faecal smears is frequently below the sensitivity threshold of the Kato-Katz faecal smear technique for direct egg detection. Several new approaches have been proposed recently to improve schistosomiasis diagnosis, via magnetic concentration techniques or field applicable flotation devices, as well as molecular and antigen-detection methods. In their contribution to this special issue, M. C. C. Espírito-Santo and colleagues review current laboratory diagnostic techniques for schistosomiasis diagnosis [4, 5].

The final adoption and deployment of diagnostics in a range of settings requires many factors to come together. The diagnostic must be technically feasible within the setting; the diagnostic must be affordable for sustained use in that setting and of course must be able to meet the demands of the many clinical and research questions asked about it. No diagnostic tool is perfect and there is ongoing demand for improvements to the current tools. The collected papers highlight a snapshot of the current state of play for diagnostics of just a few neglected tropical diseases.

*Stephan Karl* *Malcolm K. Jones* *Lucía Gutiérrez* *Brioni Moore* *Eline Kattenberg* *Marcus Lacerda*
